# Supplementary material for: The STING agonist IMSA101 enhances chimeric antigen receptor T cell function by inducing IL-18 secretion
Source: Nat Commun. 2024 May 10;15:3933. doi: 10.1038/s41467-024-47692-9 (PMC11087554; doi:10.1038/s41467-024-47692-9)
Supplement: Supplementary file 1 — Supplementary Information [file 41467_2024_47692_MOESM1_ESM.pdf]

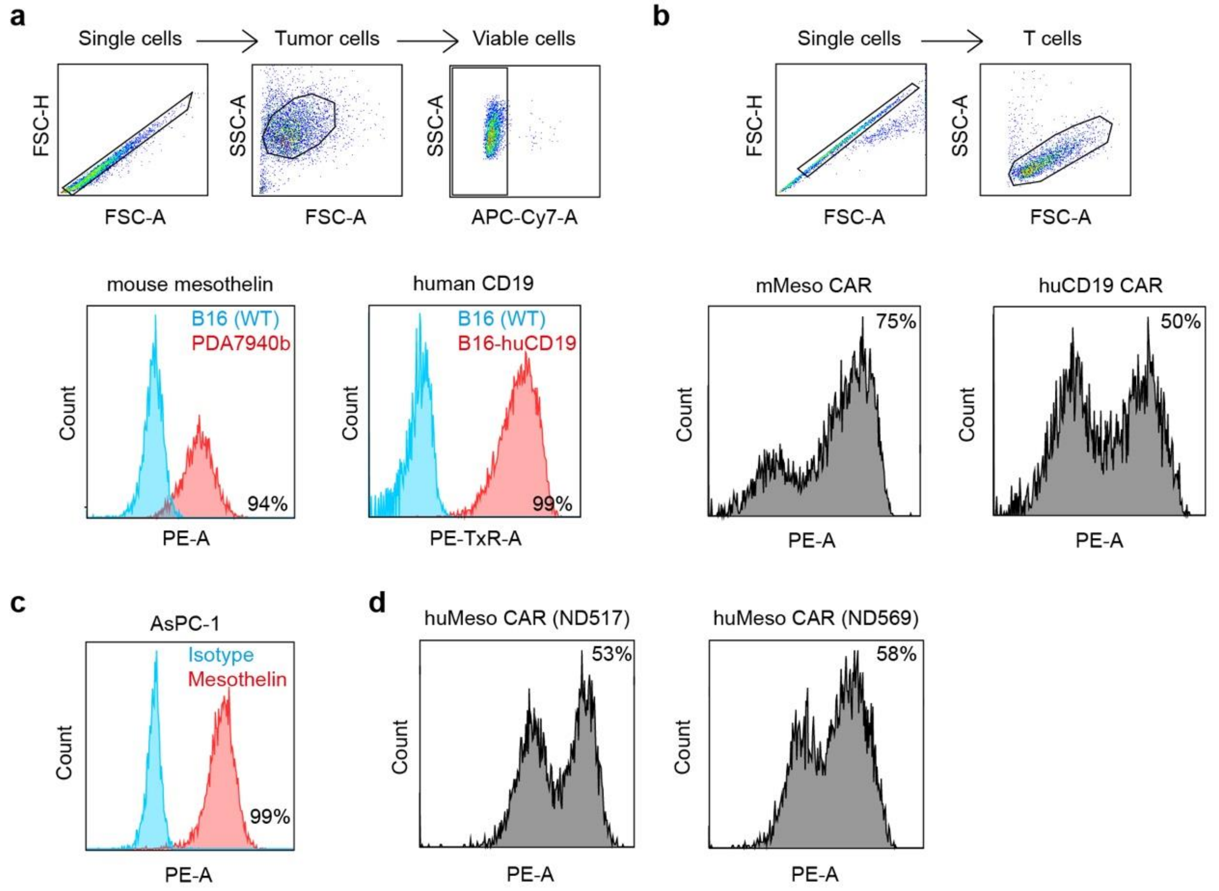

Supplementary Figure 1. **Tumor cells and T cells express the target antigens and CAR constructs in syngeneic and xenograft animal models.** **a** Murine mesothelin expression of the mouse pancreatic ductal adenocarcinoma (PDA) cell line PDA7940b, as well as human CD19 expression of the mouse melanoma cells B16-huCD19 used for syngeneic experiments in red. Staining with control cell lines are shown in blue. **b** Murine T cells were transduced to express a murine mesothelin-specific CAR construct (mMeso CAR) or a human CD19-specific CAR construct (huCD19 CAR) to be used in the PDA7940b or B16-huCD19 animal models, respectively. Shown are the rates of CAR^+ murine T cells. **c** Shows human mesothelin expression of the human PDA cell line AsPC-1. **d** Shows human mesothelin-specific CAR expression of human donors ND517 T cells (left histogram) as well as ND569 T cells (right histogram).

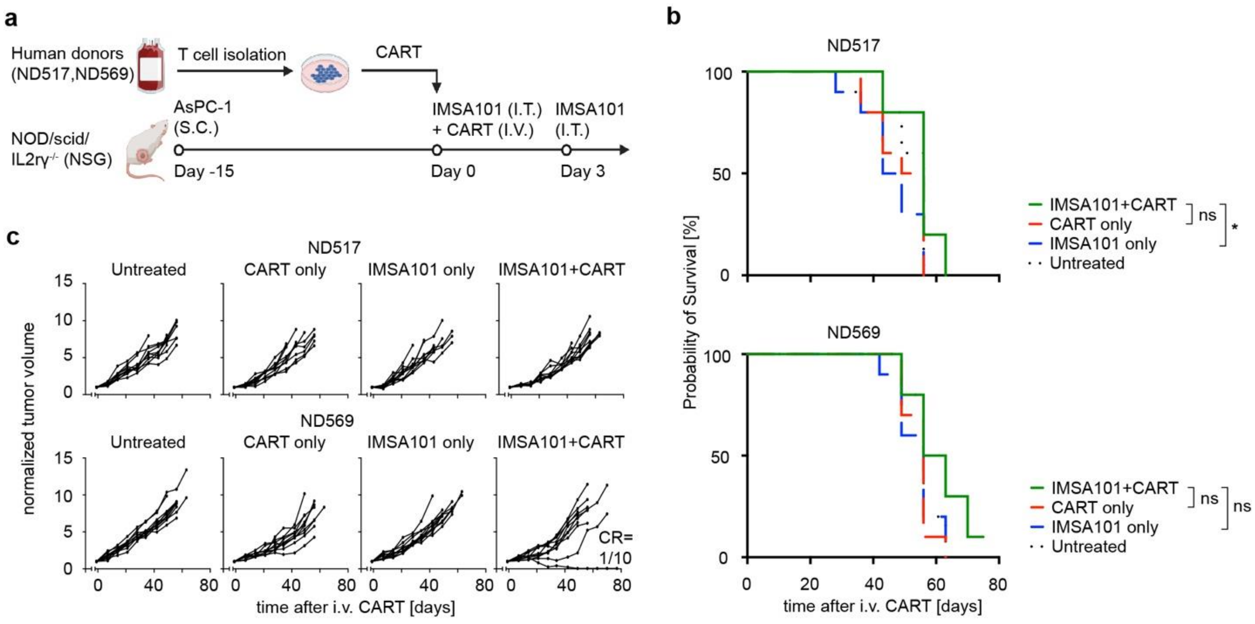

Supplementary Figure 2. **Minimal effects of IMSA101 on human CART are seen in immunodeficient NSG mice. a** Schematic of experimental design for testing the combinatorial treatment approach in immunodeficient NOD/scid/IL2rγ$^{-/-}$ (NSG) mice. Icons of this figure created with BioRender.com. **b** Kaplan-Meier survival curve (n=10 mice/cohort). Statistical significance was calculated using the log-rank Mantel-Cox test. ns (nonsignificant) $P > 0.05$; $*P \leq 0.05$. **c** Changes in tumor volume over time. n=10 mice/cohort. CR, complete remission. Source data and exact p values are provided as a Source Data file.

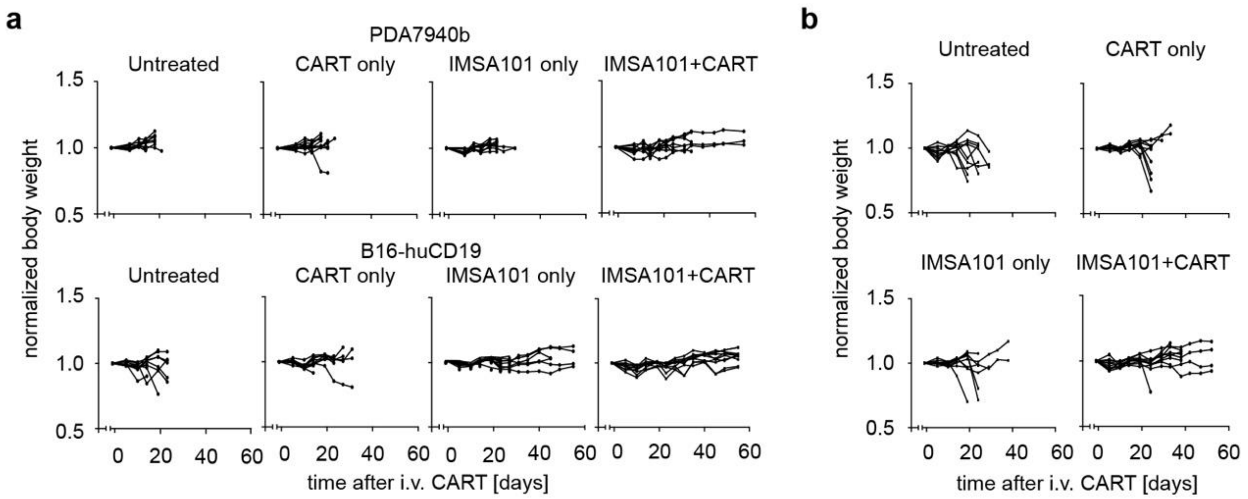

Supplementary Figure 3. **Body weight change of syngeneic mice during treatment.** Changes in body weight **a** of treated mice over time in mono-flank tumor models (PDA7940b and B16-huCD19; n=10 mice/cohort), **b** of treated mice over time in a dual-flank tumor model (B16-huCD19; n=10 mice/cohort). Values were normalized to day -1. Source data are provided as a Source Data file.

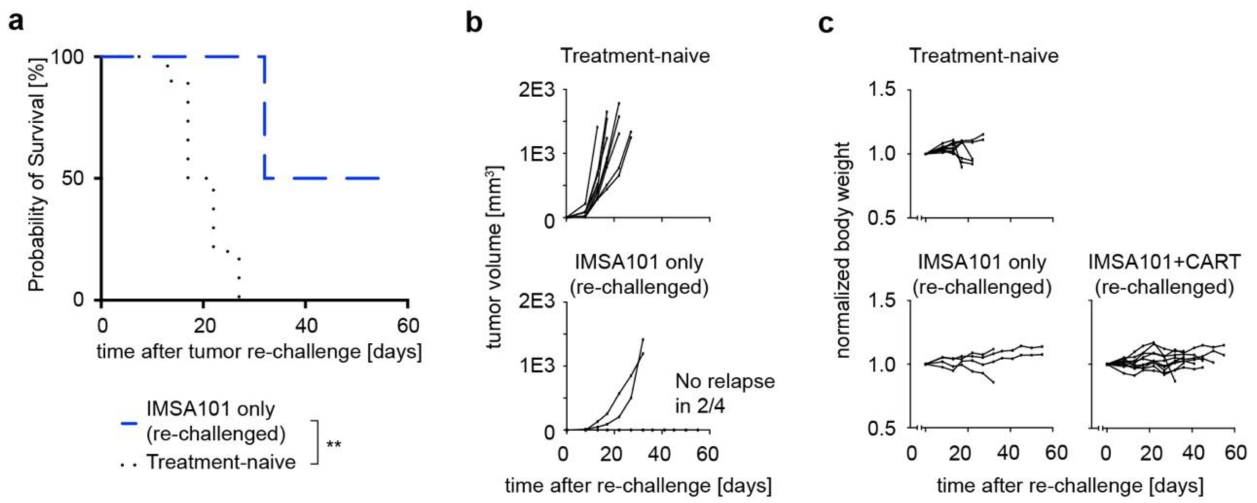

Supplementary Figure 4. **IMSA101-treated mice with complete remission show no or late tumor engraftment after tumor re-challenge.** **a** Kaplan-Meier survival curve of mice receiving tumor re-challenge with B16 cells (WT). n=4 mice (IMSA101 only) and n=10 mice (Treatment-naïve) were used. Statistical significance was calculated using the log-rank Mantel-Cox test. $^{**}P \leq 0.01$. **b** Changes in tumor volume over time. **c** Changes in body weight over time. Values were normalized to day -1. Source data and exact p values are provided as a Source Data file.

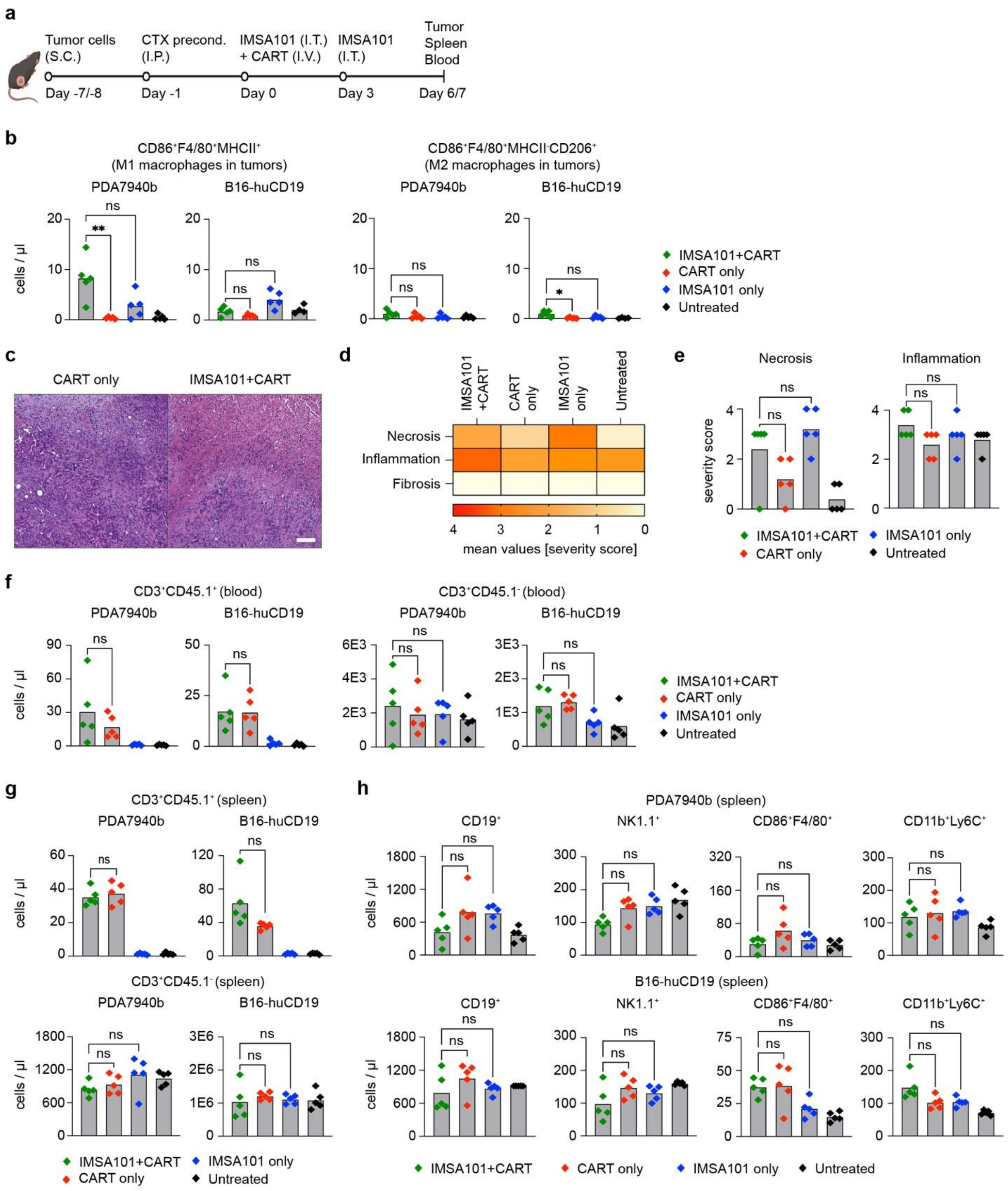

Supplementary Figure 5. **No differences between cohorts are seen in peripheral blood and spleen of immunocompetent mice. a** Schematic of experimental design to analyze changes in tumor, spleen, and peripheral blood following combinatorial treatment. Pathological assessment from tumors was performed at day 6 after treatment, and flow cytometry analyses from spleen and

peripheral blood at day 7 after treatment in separate experiments. Icons of this figure created with BioRender.com. **b** M1 and M2 macrophage count analyzed via surface staining and flow cytometry from tumor cell suspension (n=5/cohort). Average and individual values are shown. Kruskal-Wallis one-way analysis of variance was used for statistical analysis. ns (nonsignificant) $P > 0.05$; $*P \leq 0.05$; $**P \leq 0.01$. **c** One representative photomicrograph per cohort as indicated at 4X magnification of hematoxylin and eosin (H&E) staining of tumor tissues. Scale bar equal to 200μm. **d** Heatmap summarizing the pathological assessment from tumor tissues with n=5/cohort. Top box shows results of each cohort as mean values. Bottom box shows the grading scale. **e** Bar graphs summarizing changes in necrosis and inflammation as per pathological assessment (n=5/cohort). Average and individual values are shown. Kruskal-Wallis one-way analysis of variance was used for statistical analysis. ns (nonsignificant) $P > 0.05$. **f+g** $CD3^+CD45.1^+$ cell count (=infused T cells) as well as $CD3^+CD45.1^-$ cell count (= endogenous T cells) analyzed via flow cytometry from **f** peripheral blood, and **g** spleen single cell suspension (n=5/cohort). Average and individual values are shown. Kruskal-Wallis one-way analysis of variance was used for statistical analysis. ns (nonsignificant) $P > 0.05$. **h** Count of different cell populations as indicated, analyzed via flow cytometry from spleen single cell suspensions (n=5/cohort). Average and individual values are shown. Kruskal-Wallis one-way analysis of variance was used for statistical analysis. ns (nonsignificant) $P > 0.05$. Source data and exact p values are provided as a Source Data file.

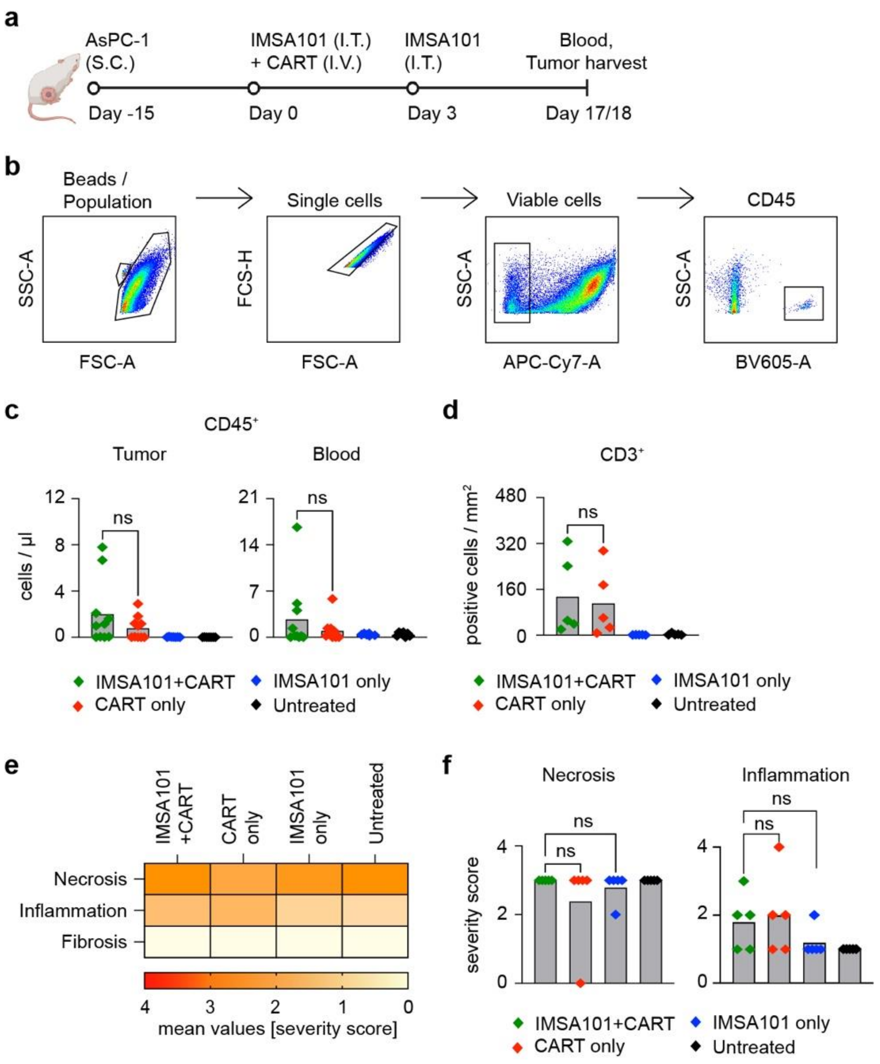

Supplementary Figure 6. **No differences between cohorts are seen in analyzed tumors and peripheral blood of immunodeficient NSG mice. a** Schematic of experimental design to analyze effects of IMSA101 on human CART. Pathological assessment from tumors was performed at day 17 after treatment, and flow cytometry analyses from tumor and spleen at day 18 after treatment in separate experiments. Icons of this figure created with BioRender.com. **b** Gating strategy for flow cytometer analyses. **c** Human $CD45^+$ cell count analyzed via flow cytometry from tumor single cell suspension and peripheral blood (n=10/cohort, pooled from both T cell donors). Average and individual values are shown. Kruskal-Wallis one-way analysis of variance was used for statistical analysis. ns (nonsignificant) P > 0.05. **d** Human $CD3^+$ cell count analyzed via IHC staining from bulk tumor tissue (n=5/cohort). Average and individual values are shown. Kruskal-Wallis one-way analysis of variance was used for statistical analysis. ns (nonsignificant) P > 0.05. **e** Heatmap summarizing the pathological assessment of tumor tissues with n=5/cohort. Top box shows results of each cohort as mean values. Bottom box shows the grading scale. **f** Bar graphs

summarizing changes in necrosis and inflammation as per pathological assessment (n=5/cohort). Average and individual values are shown. Kruskal-Wallis one-way analysis of variance was used for statistical analysis. ns (nonsignificant) $P > 0.05$. Source data and exact p values are provided as a Source Data file.

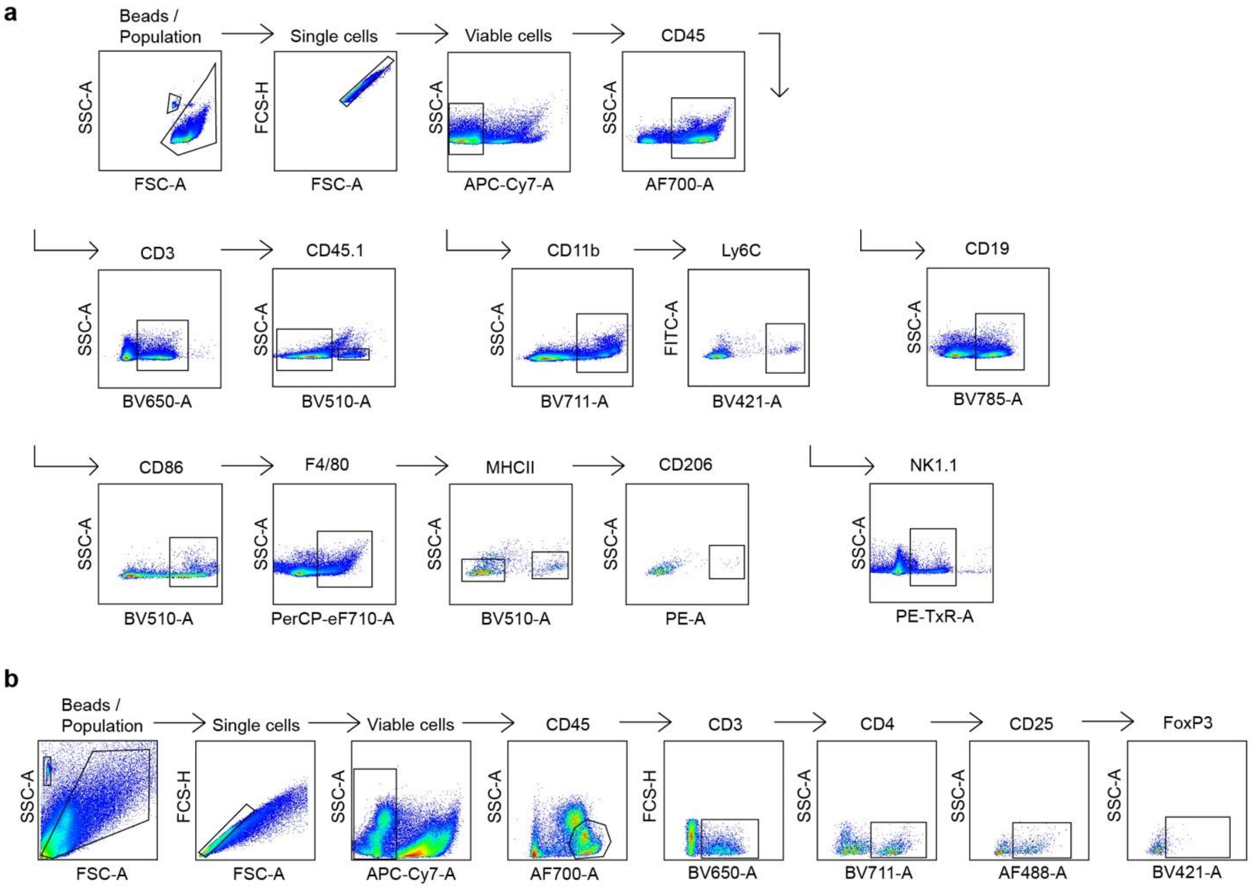

Supplementary Figure 7. **Representative gating strategy for flow cytometry analyses of immune cell populations in syngeneic mice.** Gating strategy was used to detect **a** (CAR)T cells, MDSCs, B cells, macrophages, and NK cells, as well as **b** regulatory T cells (T_{regs}).

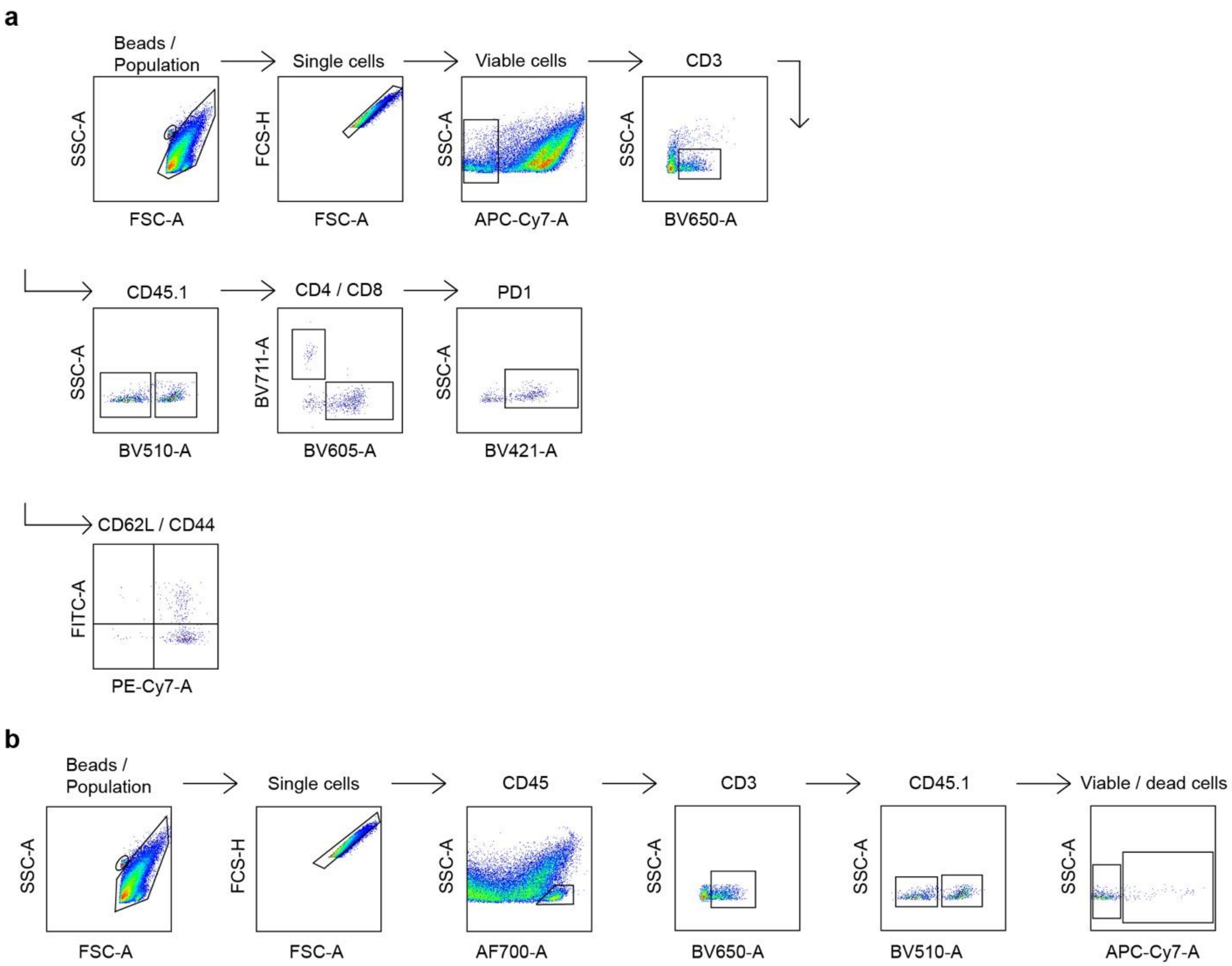

Supplementary Figure 8. **Representative gating strategy for flow cytometry analyses of intratumoral T cells in syngeneic mice.** Gating strategy was used to analyze **a** CD4/CD8 count, activation markers, and phenotype, as well as **b** viability of intratumoral T cells.

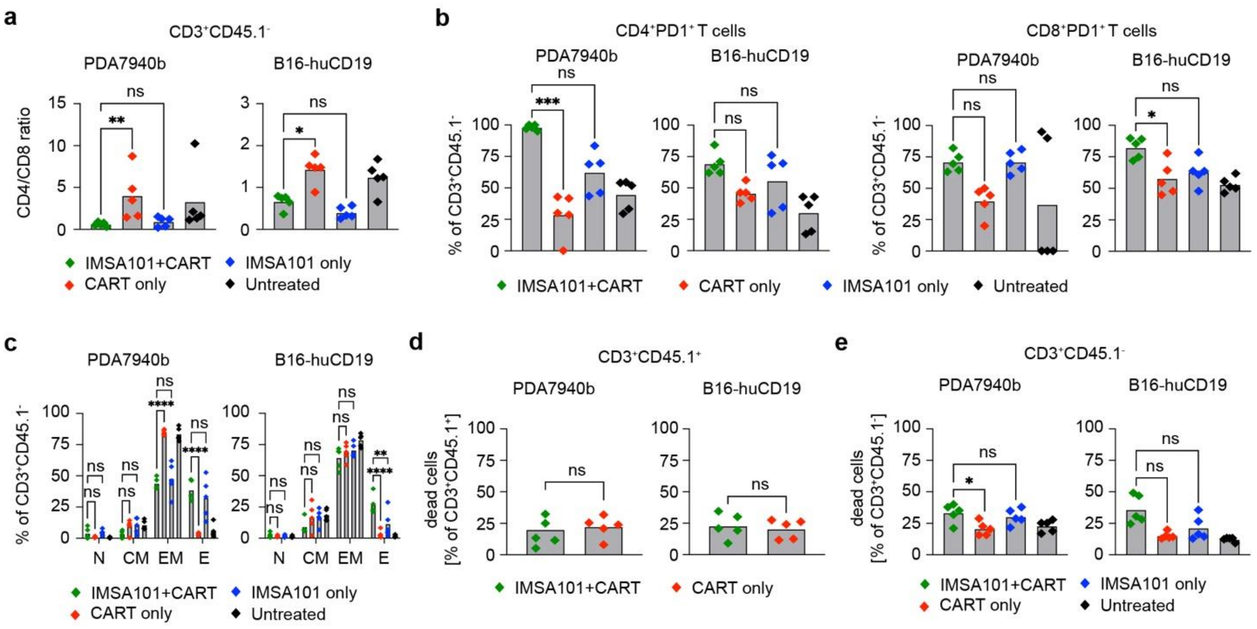

Supplementary Figure 9. **i.t. IMSA101 induces activation of intratumoral endogenous T cells.** Surface staining and flow cytometry analyses was performed from tumor single cell suspensions at day 6 after treatment in a single experiment. **a** CD4/CD8 ratio and **b** rate of PD1-expressing CD3$^+$CD45.1$^-$ cells (=endogenous T cells) are shown (n=5/cohort). Average and individual values are shown. Kruskal-Wallis one-way analysis of variance was used for statistical analysis. ns (nonsignificant) $P > 0.05$; $^*P \leq 0.05$; $^{**}P \leq 0.01$; $^{***}P \leq 0.001$. **c** Phenotype of intratumoral CD3$^+$CD45.1$^-$ cells (=endogenous T cells; n=5/cohort). N=naïve (CD44$^-$CD62L$^+$); CM=central memory (CD44$^+$CD62L$^+$); EM=effector memory (CD44$^+$CD62L$^-$); E=effector (CD44$^-$CD62L$^-$). Two-way ANOVA with Sidak's test was used for pairwise multiple comparisons. ns (nonsignificant) $P > 0.05$; $^{**}P \leq 0.01$; $^{****}P \leq 0.0001$. **d+e** rate of intratumoral dead cells (n=5/cohort) of **d** infused CAR T cells (CD3$^+$CD45.1$^+$) and **e** endogenous T cells (CD3$^+$CD45.1$^-$). Kruskal-Wallis one-way analysis of variance was used for statistical analysis. ns (nonsignificant) $P > 0.05$; $^*P \leq 0.05$. Source data and exact p values are provided as a Source Data file.

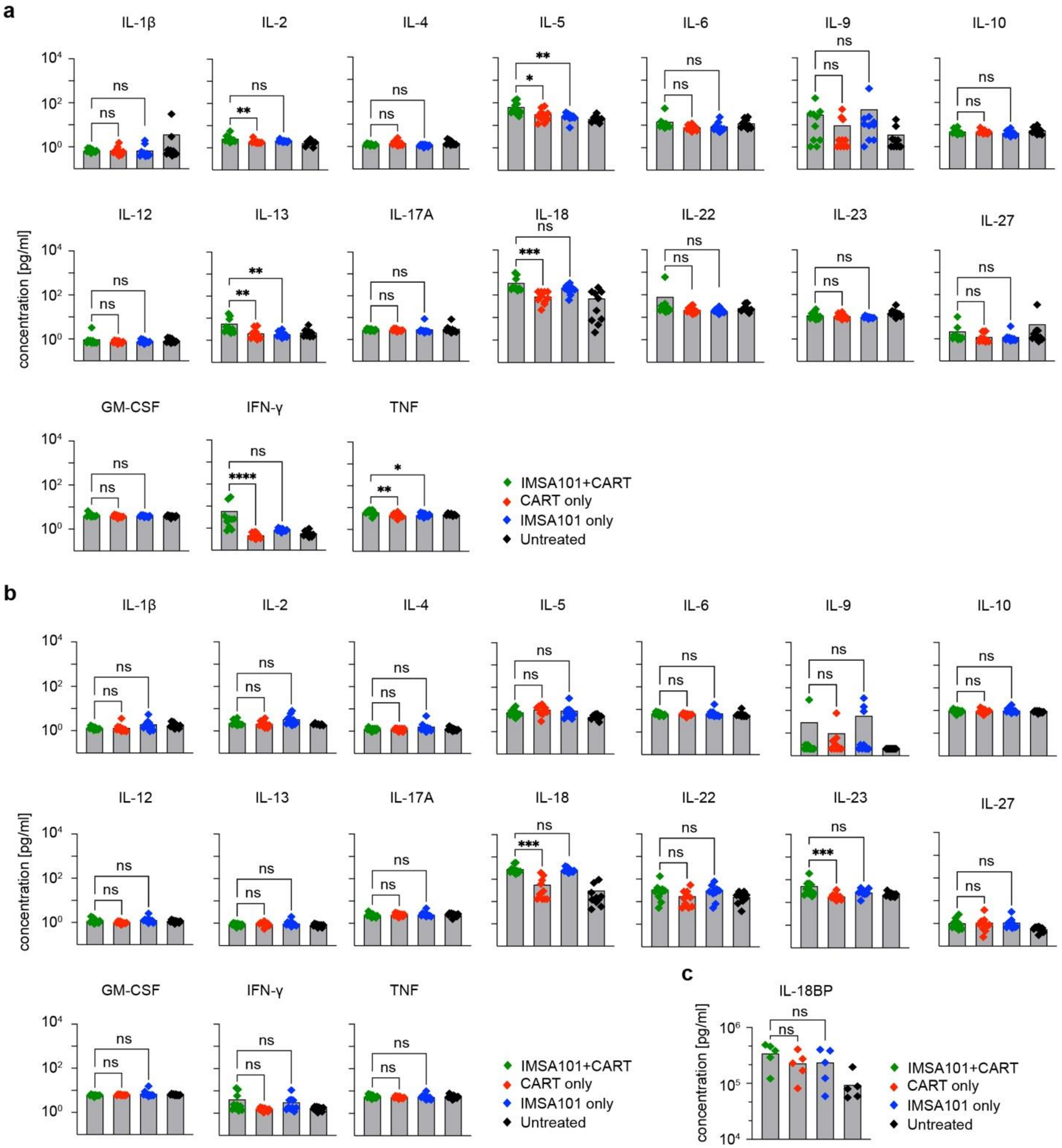

Supplementary Figure 10. **Differences between cohorts are seen in Serum cytokine levels in PDA7940b and B16-huCD19 tumor models. a-c** Cytokine concentrations in mouse serum as indicated. **a** Serum cytokine levels in PDA7940b animal model (n=10/cohort), **b** Serum cytokine levels in B16-huCD19 animal model (n=10/cohort), and **c** shows serum IL18BP levels in PDA7940b animal model (n=5/cohort). Average and individual values are shown. Kruskal-Wallis one-way analysis of variance was used for statistical analysis. ns (nonsignificant) $P > 0.05$; $*P \leq$

0.05; **$P \leq 0.01$; ***$P \leq 0.001$; ****$P \leq 0.0001$. Source data and exact p values are provided as a Source Data file.

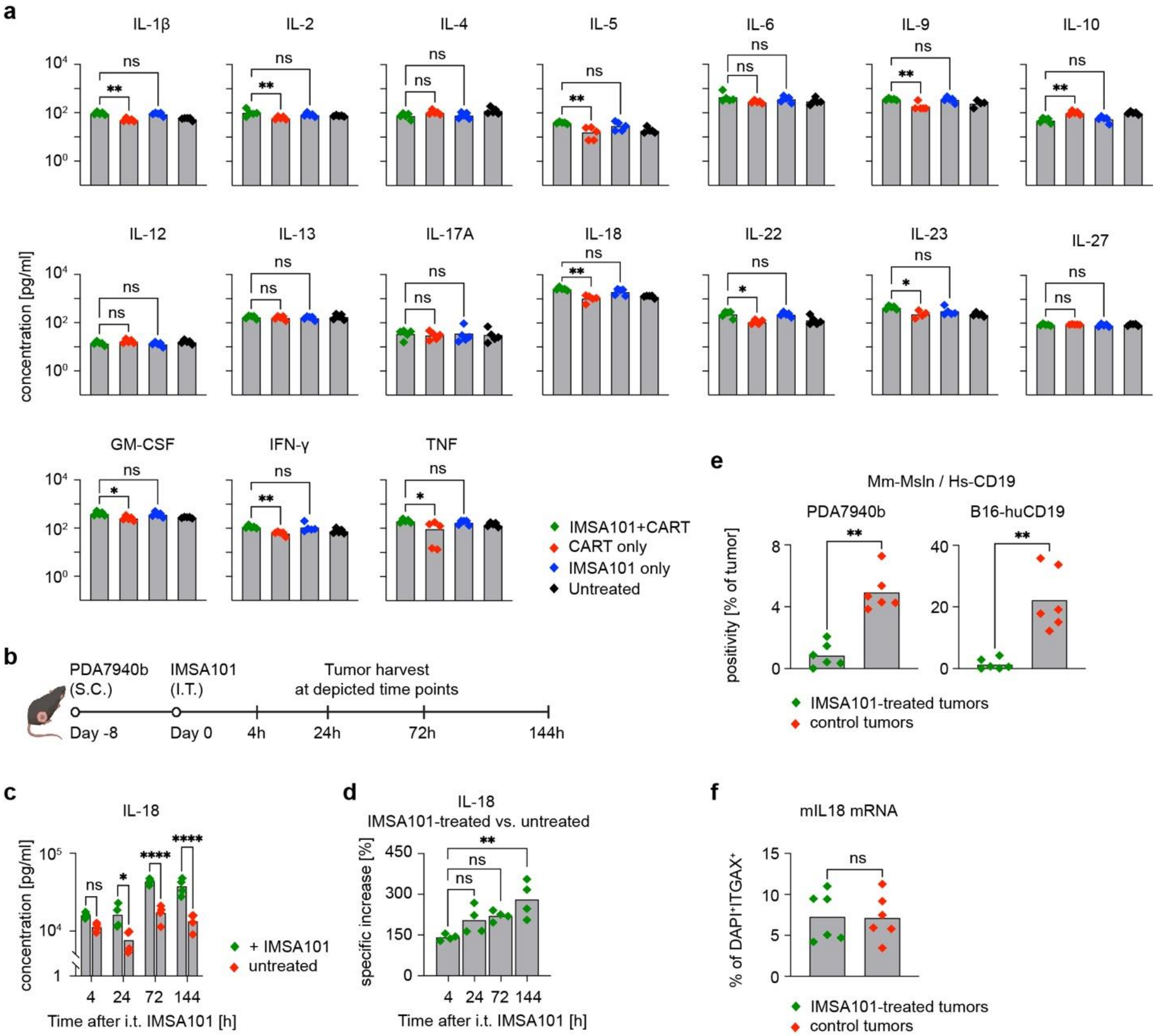

Supplementary Figure 11. **IMSA101 induces intratumoral IL-18 secretion. a** Tumor cytokine levels in PDA7940b tumor model (n=5/cohort). Experiment was performed at day 6 after treatment. Average and individual values are shown. Kruskal-Wallis one-way analysis of variance was used for statistical analysis. ns (nonsignificant) P > 0.05; *P ≤ 0.05; **P ≤ 0.01. **b-d** In vivo experiment to analyze the time-interval of IMSA101-mediated IL-18 induction. **b** Schematic of experimental design. Icons of this figure created with BioRender.com. **c** Tumor cytokine levels over time in PDA7940b tumors (n=4/cohort). Average and individual values are shown. Two-way ANOVA with Sidak's test was used for pairwise multiple comparisons. ns (nonsignificant) P > 0.05; *P ≤ 0.05; ****P ≤ 0.0001. **d** Shows rate of specific IL-18 increase in IMSA101-treated tumors (n=4/cohort). Average IL-18-levels in untreated tumors was used as baseline to compare specific increase in IMSA1010-treated tumors. Average and individual values are shown. Kruskal-

Wallis one-way analysis of variance was used for statistical analysis. ns (nonsignificant) $P > 0.05$; $^{**}P \leq 0.01$. **e** Shows bar graphs summarizing detectable Mm-Msln/Hs-CD19 RNA in tumor samples using RNA-ISH (n=6/cohort). Average and individual values are shown. Mann-Whitney U Test was used for statistical analysis. ns (nonsignificant) $P > 0.05$; $^{**}P \leq 0.01$. **f** Rate of detectable Mm-IL-18 RNA in $DAPI^{+}ITGAX^{+}$ cells of PDA7940b tumor samples (n=6/cohort). Average and individual values are shown. Mann-Whitney U Test was used for statistical analysis. ns (nonsignificant) $P > 0.05$. Source data and exact p values are provided as a Source Data file.

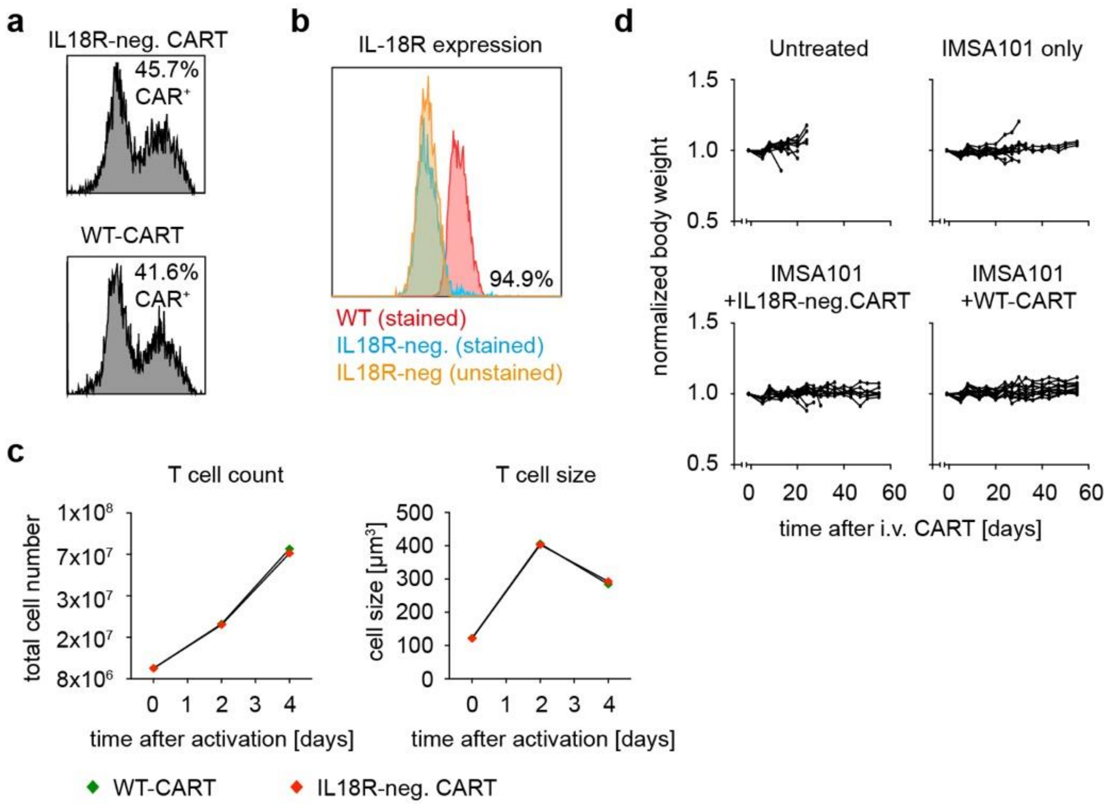

Supplementary Figure 12. **Expansion and CAR-transduction rate of wild type and IL-18 receptor negative T cells. a** Murine T cells, either wild type (WT-CART) or IL-18 receptor negative (IL18R-neg. CART), were transduced to express a human CD19-specific CAR construct to be used in the B16-huCD19 animal model. Shown is the rate of CAR^+ murine IL18R-neg. CART and CAR^+ WT-CART prior infusion into mice. **b** Shows IL-18R expression of WT-CART as well as IL18R-neg. CART prior infusion into mice. **c** Shows cell count and cell size during manufacturing of WT-CART as well as IL18R-neg. CART from mouse T cells of one representative experiment. **d** Changes in body weight of treated mice over time (n=12 mice/cohort). Values were normalized to day -1. Source data are provided as a Source Data file.
