# Supplementary material for: The STING agonist IMSA101 enhances chimeric antigen receptor T cell function by inducing IL-18 secretion
Source: Nat Commun. 2024 May 10;15:3933. doi: 10.1038/s41467-024-47692-9 (PMC11087554; doi:10.1038/s41467-024-47692-9)
Supplement: Supplementary file 3 — Description of Additional Supplementary Files [file 41467_2024_47692_MOESM3_ESM.pdf]

Description of Additional Supplementary Files

Supplementary Data 1: Overview of Log Fold Change (LFC) and negative log of adjusted p-values (nlog10pval) of differentially expressed genes (DEGs).

Supplementary Data 2: Overview of Log Fold Change (LFC) and negative log of adjusted p-values (nlog10pval) of Gene Set Variance Analyses (GSVA).

Supplementary Data 3: Normalized counts of full NanoString nCounter dataset.
