# Supplementary material for: The STING agonist IMSA101 enhances chimeric antigen receptor T cell function by inducing IL-18 secretion
Source: Nat Commun. 2024 May 10;15:3933. doi: 10.1038/s41467-024-47692-9 (PMC11087554; doi:10.1038/s41467-024-47692-9)
Supplement: Supplementary file 7 — Reporting Summary [file 41467_2024_47692_MOESM7_ESM.pdf]

nature portfolio

Corresponding author(s): Carl H. June, MD
Zhijian J. Chen, PhD

Last updated by author(s): 03/29/2024

Reporting Summary

Nature Portfolio wishes to improve the reproducibility of the work that we publish. This form provides structure for consistency and transparency in reporting. For further information on Nature Portfolio policies, see our Editorial Policies and the Editorial Policy Checklist.

Statistics

For all statistical analyses, confirm that the following items are present in the figure legend, table legend, main text, or Methods section.

n/a	Confirmed	
☐	☒	The exact sample size (*n*) for each experimental group/condition, given as a discrete number and unit of measurement
☐	☒	A statement on whether measurements were taken from distinct samples or whether the same sample was measured repeatedly
☐	☒	The statistical test(s) used AND whether they are one- or two-sided<br>*Only common tests should be described solely by name; describe more complex techniques in the Methods section.*
☒	☐	A description of all covariates tested
☒	☐	A description of any assumptions or corrections, such as tests of normality and adjustment for multiple comparisons
☐	☒	A full description of the statistical parameters including central tendency (e.g. means) or other basic estimates (e.g. regression coefficient) AND variation (e.g. standard deviation) or associated estimates of uncertainty (e.g. confidence intervals)
☐	☒	For null hypothesis testing, the test statistic (e.g. *F*, *t*, *r*) with confidence intervals, effect sizes, degrees of freedom and *P* value noted<br>*Give P values as exact values whenever suitable.*
☒	☐	For Bayesian analysis, information on the choice of priors and Markov chain Monte Carlo settings
☒	☐	For hierarchical and complex designs, identification of the appropriate level for tests and full reporting of outcomes
☒	☐	Estimates of effect sizes (e.g. Cohen's *d*, Pearson's *r*), indicating how they were calculated

Our web collection on statistics for biologists contains articles on many of the points above.

Software and code

Policy information about availability of computer code

Data collection	BD FACSDiva (v6.1.2)
Data analysis	GraphPad Prism version 9 (GraphPad Software), Adobe Illustrator (Adobe), BioRender under paid license (https://biorender.com), nSolver Analysis software, ggplot2 Bioconductor R package, FlowJo (v10.7.1)

For manuscripts utilizing custom algorithms or software that are central to the research but not yet described in published literature, software must be made available to editors and reviewers. We strongly encourage code deposition in a community repository (e.g. GitHub). See the Nature Portfolio guidelines for submitting code & software for further information.

Data

Policy information about availability of data

All manuscripts must include a data availability statement. This statement should provide the following information, where applicable:
- Accession codes, unique identifiers, or web links for publicly available datasets
- A description of any restrictions on data availability
- For clinical datasets or third party data, please ensure that the statement adheres to our policy

The authors declare that all data of this study are available within the article, Supplementary Information file, Supplementary Data files, or Source Data file. Source data are provided with this paper. Supplementary Data are also available in Figshare [https://doi.org/10.6084/m9.figshare.24183867].

Research involving human participants, their data, or biological material

Policy information about studies with human participants or human data. See also policy information about sex, gender (identity/presentation), and sexual orientation and race, ethnicity and racism.

Reporting on sex and gender	n/a
Reporting on race, ethnicity, or other socially relevant groupings	n/a
Population characteristics	n/a
Recruitment	n/a
Ethics oversight	n/a

Note that full information on the approval of the study protocol must also be provided in the manuscript.

Field-specific reporting

Please select the one below that is the best fit for your research. If you are not sure, read the appropriate sections before making your selection.

☒ Life sciences ☐ Behavioural & social sciences ☐ Ecological, evolutionary & environmental sciences

For a reference copy of the document with all sections, see nature.com/documents/nr-reporting-summary-flat.pdf

Life sciences study design

All studies must disclose on these points even when the disclosure is negative.

Sample size	For in vivo experiment, a minimum of 10 animals/cohort were used as depicted in figure legends. Sample sizes were not determined using staistical methods, but based on extensive experience in the literature (including from our group) using PDA/B16 mouse flank tumor models and CAR T cells. Please also see following publications: DOI: 10.1038/s41586-022-04801-2 ; DOI: 10.1016/j.cell.2021.08.004 ; DOI: 10.1016/j.cell.2021.11.016.
Data exclusions	No data were excluded from the analysis.
Replication	Mouse experiments were at least performed twice to verify reproducibility, except of gene expression/pathway enrichment analysis, flow cytometry of single cell suspensions, IHC/pathology, RNA-ISH, and cytokine analysis experiments. In vitro experiments were performed in technical triplicates. For gene expression/pathway analysis, technical duplicates were used. For Luminex cytokine analysis, flow cytometry of single cell suspension, RNA-ISH, and IHC/pathology at least four biological replicates were used as depicted in figure legends. All attempts at replication were successful.
Randomization	For all mouse experiments, mice were selected based on tumor size and groups were generated randomly. For other experiments, samples were randomly allocated into experimental groups.
Blinding	For all experiments, investigators were blinded to group allocation during data collection and analysis.

Reporting for specific materials, systems and methods

We require information from authors about some types of materials, experimental systems and methods used in many studies. Here, indicate whether each material, system or method listed is relevant to your study. If you are not sure if a list item applies to your research, read the appropriate section before selecting a response.

Materials & experimental systems

n/a	Involved in the study	
☐	☒	Antibodies
☐	☒	Eukaryotic cell lines
☒	☐	Palaeontology and archaeology
☐	☒	Animals and other organisms
☒	☐	Clinical data
☒	☐	Dual use research of concern
☒	☐	Plants

Methods

n/a	Involved in the study	
☒	☐	ChIP-seq
☐	☒	Flow cytometry
☒	☐	MRI-based neuroimaging

Antibodies

Antibodies used	The following antibodies were used for this study:<br>1) Biotin anti-human mesothelin (1/25 dilution; clone MB; CAT: 530203; BioLegend), lot: B334484<br>2) Biotin mouse IgG2a, κ isotype ctrl (1/25 dilution; clone MOPC-173; CAT: 400203; BioLegend), lot: B246068<br>3) PE/Dazzle 594 anti-human CD19 (1/100 dilution; clone HIB19; CAT: 302252; Biolegend), lot: B358189<br>4) Monoclonal rat anti-mouse MSLN / Mesothelin (1/25 dilution; clone: B35; CAT: LS-C179484; LSBio), lot: 220300<br>5) PE mouse anti-rat IgG2a (1/100 dilution; clone: r2a-21B2; CAT: 12-4817-82; Invitrogen), lot: 2560184<br>6) Brilliant Violet 605 anti-human CD45 antibody (1/80 dilution; clone 2D1; CAT: 368524; BioLegend), lot: B366217<br>7) Alexa Fluor 700 anti-mouse CD45 (1/200 dilution; clone 30-F11; CAT: 103128; BioLegend), lot: B358309<br>8) Brilliant Violet 650 anti-mouse CD3 (1/40 dilution; clone 17A2; CAT: 100229; BioLegend), lot: B350667<br>9) Brilliant Violet 510 anti-mouse CD45.1 (1/50 dilution; clone A20; CAT: 110741; BioLegend), lot: B359883<br>10) Brilliant Violet 785 anti-mouse CD19 (1/50 dilution; clone 6D5; CAT: 115543; BioLegend), lot: B337740<br>11) PE/Dazzle 594 anti-mouse NK1.1 (1/100 dilution; clone PK136; CAT: 108748; BioLegend), lot: B354066<br>12) Alexa Fluor 647 anti-mouse CD86 (1/100 dilution; clone GL-1; CAT: 105020; BioLegend), lot: B357819<br>13) PerCP/Cyanine 5.5 anti-mouse F4/80 (1/50 dilution; clone BM8; CAT: 123128; BioLegend), lot: B347137<br>14) Brilliant Violet 510 anti-mouse I-A/I-E (1/100 dilution; clone M5/114.15.2; CAT: 107636; BioLegend), lot: B363170<br>15) Brilliant Violet 711 anti-mouse/human CD11b (1/100 dilution; clone M1/70; CAT: 101242,;BioLegend), lot: B345610<br>16) Brilliant Violet 421 anti-mouse Ly-6C (1/50 dilution; clone HK1.4; CAT: 128032; BioLegend), lot: B348618<br>17) Alexa Fluor 488 anti-mouse CD25 (1/100 dilution; clone PC61; CAT: 102017; BioLegend), lot: B245907<br>18) Brilliant Violet 421 anti-mouse FoxP3 (1/100 dilution; clone MF-14; CAT: 126419; BioLegend), lot: B285905<br>19) Brilliant Violet 421 Rat IgG2b Isotype Ctrl (1/100 dilution; clone RTK4530; CAT: 400639; Biolegend), lot: B402134<br>20) Brilliant Violet 711 anti-mouse CD4 (1/200 dilution; clone RM4-5; CAT: 100550; BioLegend), lot: B340491<br>21) Brilliant Violet 605 anti-mouse CD8a (1/160 dilution; clone 53-6.7; CAT: 100744; BioLegend), lot: B360994<br>22) Brilliant Violet anti-mouse CD279/PD-1 (1/50 dilution; clone 29F.1A12; CAT: 135218; BioLegend), lot: B349792<br>23) PE/Cyanine7 anti-mouse/anti-human CD44 (1/100 dilution; clone IM7; CAT: 103030; BioLegend), lot: B345004<br>24) FITC anti-mouse CD62L (1/100 dilution; clone MEL-14; CAT: 104406; BioLegend), lot: B338689<br>25) Biotin-SP (long spacer) AffiniPure F(ab')$_2$ fragment goat anti-human IgG (1/25 dilution; CAT: 109-066-006; Jackson ImmunoResearch), lot: 163828<br>26) APC anti-mouse CD218a (IL-18Rα) (1/100 dilution; clone A17071D; CAT: 157906; BioLegend), lot: B379723<br>27) PE Streptavidin (1/100 dilution; CAT: 554061; BD Biosciences), lot: 1057256
Validation	All antibodies used are commercially available and validation is noted on the manufacturer's website as follows:<br>ad1) https://www.biolegend.com/nl-nl/products/biotin-anti-human-mesothelin-antibody-12959<br>ad2) https://www.biolegend.com/de-at/products/biotin-mouse-igg2a-kappa-isotype-ctrl-1398<br>ad3) https://www.biolegend.com/en-gb/products/pe-dazzle-594-anti-human-cd19-antibody-9783<br>ad4) https://www.lsbio.com/antibodies/msln-antibody-mesothelin-antibody-clone-b35-flow-ls-c179484/186958<br>ad5) https://www.thermofisher.com/antibody/product/Mouse-anti-Rat-IgG2a-Secondary-Antibody-clone-r2a-21B2-Monoclonal/12-4817-82<br>ad6) https://www.biolegend.com/en-ie/products/brilliant-violet-605-anti-human-cd45-antibody-14717?GroupID=BLG14850<br>ad7) https://www.biolegend.com/en-ie/products/alexa-fluor-700-anti-mouse-cd45-antibody-3407?GroupID=BLG6833<br>ad8) https://www.biolegend.com/en-gb/products/brilliant-violet-650-anti-mouse-cd3-antibody-7843?GroupID=BLG242<br>ad9) https://www.biolegend.com/fr-fr/products/brilliant-violet-510-anti-mouse-cd45-1-antibody-9609<br>ad10) https://www.biolegend.com/fr-lu/products/brilliant-violet-785-anti-mouse-cd19-antibody-7962<br>ad11) https://www.biolegend.com/en-gb/products/pe-dazzle-594-anti-mouse-nk-1-1-antibody-10320?GroupID=GROUP20<br>ad12) https://www.biolegend.com/ja-jp/products/alexa-fluor-647-anti-mouse-cd86-antibody-3121<br>ad13) https://www.biolegend.com/nl-be/products/percp-cyanine5-5-anti-mouse-f480-antibody-4303?GroupID=BLG5319<br>ad14) https://www.biolegend.com/en-ie/products/brilliant-violet-510-anti-mouse-i-a-i-e-antibody-7997?GroupID=BLG11931<br>ad15) https://www.biolegend.com/fr-ch/products/brilliant-violet-711-anti-mouse-human-cd11b-antibody-7927?GroupID=BLG10552<br>ad16) https://www.biolegend.com/en-ie/products/brilliant-violet-421-anti-mouse-ly-6c-antibody-8586?GroupID=BLG5853<br>ad17) https://www.biolegend.com/nl-be/products/alexa-fluor-488-anti-mouse-cd25-antibody-2706<br>ad18) https://www.biolegend.com/en-gb/products/brilliant-violet-421-anti-mouse-foxp3-antibody-12143?GroupID=BLG5706<br>ad19) https://www.biolegend.com/ja-jp/products/brilliant-violet-421-rat-igg2b-kappa-isotype-ctrl-7136?GroupID=ImportedGROUP1<br>ad20) https://www.biolegend.com/fr-ch/products/brilliant-violet-711-anti-mouse-cd4-antibody-7925<br>ad21) https://www.biolegend.com/en-gb/sean-tuckers-tests/brilliant-violet-605-anti-mouse-cd8a-antibody-7636<br>ad22) https://www.biolegend.com/de-at/products/brilliant-violet-421-anti-mouse-cd279-pd-1-antibody-7330?GroupID=BLG7927<br>ad23) https://www.biolegend.com/nl-be/products/pe-cyanine7-anti-mouse-human-cd44-antibody-3932<br>ad24) https://www.biolegend.com/fr-lu/products/fitc-anti-mouse-cd62l-antibody-384?GroupID=BLG10714<br>ad25) https://www.jacksonimmuno.com/catalog/products/109-066-006<br>ad26) https://www.biolegend.com/ja-jp/products/apc-anti-mouse-cd218a-il-18ralpha-antibody-19416<br>ad27) https://www.bdbiosciences.com/en-us/products/reagents/flow-cytometry-reagents/research-reagents/single-color-antibodies-ruo/pe-streptavidin.554061

Eukaryotic cell lines

Policy information about cell lines and Sex and Gender in Research

Cell line source(s)	B16-F10: ATCC; PDA7940b: Dr. Gregory Beatty, University of Pennsylvania; PLAT-E: Cell Biolabs; HEK293T: ATCC; AsPC-1:

	ATCC
Authentication	Cell lines were periodically authenticated by the University of Arizona Genetics Core using Promega PowerPlex16HS assay.
Mycoplasma contamination	Cell lines were tested negative in regular intervals for the presence of mycoplasma contamination by the Department of Genetics at the University of Pennsylvania (MycoAlert Mycoplasma Detection Kit, Lonza).
Commonly misidentified lines (See ICLAC register)	No commonly misidentified lines were used.

Animals and other research organisms

Policy information about studies involving animals; ARRIVE guidelines recommended for reporting animal research, and Sex and Gender in Research

Laboratory animals	NSG mice were originally procured from Jackson Laboratories and bred by the Stem Cell & Xenograft Core (SCXC) at University of Pennsylvania. For syngeneic mouse experiments, C57BL/6 mice, B6.SJL-Ptprca Pepcb/BoyJ mice, and B6.129P2-IL18r1tm1Aki/J mice were obtained from Jackson Laboratories. Six- to eight-week-old female mice were used for in vivo experiments. Mice were maintained under pathogen free conditions. Mice were subject to routine veterinary assessment for signs of overt illness and were euthanized at experimental termination or when predetermined IACUC rodent health endpoints were reached.
Wild animals	The study did not involve wild animals.
Reporting on sex	Based on extensive experience in the literature (including from our group) using PDA/B16 mouse flank tumor models and CAR T cells, sex-based analyses were not performed in the current study.
Field-collected samples	The study did not involve samples collected from the field.
Ethics oversight	The University of Pennsylvania Institutional Animal Care and Use Committee (IACUC) approved all animal experiments (protocol number: 804226), and all animal procedures were performed in the animal facility at the University of Pennsylvania in accordance with Federal and Institutional IACUC requirements.

Note that full information on the approval of the study protocol must also be provided in the manuscript.

Plants

Seed stocks	n/a
Novel plant genotypes	n/a
Authentication	n/a

Flow Cytometry

Plots

Confirm that:

- [x] The axis labels state the marker and fluorochrome used (e.g. CD4-FITC).
- [x] The axis scales are clearly visible. Include numbers along axes only for bottom left plot of group (a 'group' is an analysis of identical markers).
- [x] All plots are contour plots with outliers or pseudocolor plots.
- [x] A numerical value for number of cells or percentage (with statistics) is provided.

Methodology

Sample preparation	Source: primary mouse or human cells in culture; single cell suspensions of mouse tumors after enzymatic digestion; single cell suspension of mouse spleen after mechanical dissociation, peripheral blood from cardiac puncture. Detailed sample preparation is listed in the methods section of the manuscript.
Instrument	All data were collected by a LSRFortessa cytometer (BD Biosciences).
Software	FACSDiva software (BD Biosciences), FlowJo (v10.7.1)

Cell population abundance	n/a
Gating strategy	For all analysis, dead cells and debris were gated out using FSC-A/SSC-A and viability staining. Singlets were gated based on FSC-A and FSC-H. For subsequent gating, populations (e.g. CD45+, CD3+, ect.) were gated based on distinct expression of relevant markers. See also representative gating strategy in the Supplementary Information of the manuscript.

☒ Tick this box to confirm that a figure exemplifying the gating strategy is provided in the Supplementary Information.
